# Experimental Evaluation of the Effects of Discrete-Grading-Induced Discontinuities on the Material Properties of Functionally Graded Ti-6Al-4V Lattices

**DOI:** 10.3390/ma17040822

**Published:** 2024-02-08

**Authors:** Junyang Ye, Ata Babazadeh-Naseri, C. Fred Higgs III, Benjamin J. Fregly

**Affiliations:** Department of Mechanical Engineering, Rice University, Houston, TX 77005, USA; jy76@rice.edu (J.Y.); ata.babazadeh@gmail.com (A.B.-N.); higgs@rice.edu (C.F.H.I.)

**Keywords:** hierarchical lattices, manufacturing defects, digital image correlation, experimental, functionally graded lattice, micro-CT, Ti-6Al-4V, selective laser melting

## Abstract

In this study, we compared the material properties of linearly and sharply graded Ti6Al4V additively manufactured samples to investigate whether the more severe discontinuities caused by sharp grading can reduce performance. We performed compression testing with digital image correlation (DIC) in two loading directions for each grading design to simulate iso-stress and iso-strain conditions. We extracted the elastic stiffness, yield strength, yield strain, and energy absorption capacity of each sample. In addition, we used micro-computed tomography (micro-CT) imaging to examine the printing quality and dimensional accuracy. We found that sharply graded struts have a 12.95% increase in strut cross-sectional areas, whereas linearly graded struts produced an average of 49.24% increase compared to design. However, sharply graded and linearly graded FGL samples do not have statistically significant differences in elastic stiffness and yield strength. For the iso-strain condition, the average DIC-corrected stiffnesses for linearly and sharply graded samples were 6.15 GPa and 5.43 GPa, respectively (*p* = 0.4466), and the yield stresses were 290.4 MPa and 291.2 MPa, respectively (*p* = 0.5734). Furthermore, we confirmed different types of printing defects using micro-CT, including defective pores and disconnected struts. These results suggest that the loss of material properties caused by manufacturing defects outweighs the adverse effects of discrete-grading-induced discontinuities.

## 1. Introduction

Recent developments in additive manufacturing (AM) and selective laser melting (SLM) using biocompatible Ti-6Al-4V (Ti64) powder have expanded the applications of metal AM to medical implants. This advancement is primarily attributed to AM’s capability to produce complex geometries that were not possible using conventional subtractive manufacturing [1,2,3,4]. Additively manufactured patient-specific medical devices have the benefit of matching individual patient’s bone anatomy due to the high customizability of AM process [5,6,7]. Additionally, unlike subtractive manufacturing, where most products have solid internals [8], additive manufacturing can accommodate intricate micro-architected internal geometry [9]. This feature is particularly desirable for designing and manufacturing human bone implants because of the tunable material properties of the implants, made possible by functionally graded lattices (FGLs) [10]. Implants that incorporate FGLs can closely match bone properties [11], including bone stiffness [12] and anisotropy [13], while also maintaining their biocompatibility [14,15]. Mimicking bone properties addresses the challenge of stress shielding, where the implant’s stiffness exceeds that of the bone, leading to potential implant loosening and bone resorption [16]. In addition, additively manufactured implants introduce deliberate porosity and surface roughness via implementing FGLs to facilitate bone ingrowth and enhanced fixation due to improved osseointegration [17]. However, robust grading of truss-based FGLs is non-trivial and requires special considerations for efficient computational modeling, robust design optimization, and manufacturing constraints.

Past research has proposed various methods of functional grading for truss-based lattice structures. One approach is the unit cell size grading, which adjusts the overall size of the unit cell by scaling unit cells proportionally [18]. The solid volume fractions (*VF_s_*) of layers containing unit cells of different sizes are always constant. With no control over the *VF_s_*, unit cell size grading provides little tunability over the effective material properties of the resulting FGLs. In addition, this method creates challenges in blending unit cell layers in a 3D space due to inconsistencies between the gridded structures of different unit cell sizes. An alternative approach is adjusting the aspect ratio of unit cells, allowing them to expand or shrink in specific dimensions while keeping the others constant, thus deviating from the standard cubic grid [19]. Nonetheless, this technique shows limited success with truss-based lattices as the *VF_s_* range for different graded layers is minimal. The most prevalent method is density grading, which changes the strut thickness within unit cells, thus achieving a larger tunable range of *VF_s_* while preserving the grid structure. Some researchers adopted density grading by continuously changing the strut thickness even within individual unit cells [20,21], which has its own downsides. Notably, computational modeling of continuously graded lattices demands direct modeling of the entire structure due to the lack of clear representative volume elements (RVEs). The lack of RVEs can cause challenges in employing standard multiscale simulation methods since the inherent assumptions of periodicity and separation of scales that come with these methods are no longer valid in continuously graded FGLs. Additionally, optimizing continuously graded FGLs becomes complex, given the vastness of continuous parameters in a multi-dimensional space.

To save the computational analysis cost by using RVEs, researchers have developed a new density grading method using the hierarchical design concept. This method builds complex FGLs by assembling pre-determined basic unit cells of different solid volume fractions and orientations into the standard cubic grid. Discrete unit cell-based density grading shares the benefit of a large tunable solid volume fraction range with its continuous density grading counterpart, but it is also easily modeled using multiscale finite element (FE) methods and homogenization techniques [22,23,24]. Discretely graded lattice structures based on the hierarchical design concept are also more robust in optimization due to a reduced parameter space. Because of these reasons, discretely graded lattice structures using the hierarchical method have been used extensively in previous studies. For instance, Liu et al. chose the hierarchical FGL as a basis for their data-driven approach to designing graded composite lattice structures [25]. Wang et al. explored novel ways of designing non-uniform lattice structures using hierarchical building blocks (or basic unit cells) to achieve superior mechanical performance at an affordable computational cost [26]. Cheng et al. presented a geometric design method for a multilevel lattice structure based on hierarchical unit cells and performed finite element analysis on a lattice-filled orthopedic molar, which obtained the required material properties [27]. Gu et al. proposed a new approach to designing hierarchical materials using machine learning trained with a database of hundreds of thousands of structures from finite element analysis [28]. However, despite the advantages of hierarchical FGLs in modeling efficiency and optimizing stability, the use of discrete unit cells inevitably leads to discontinuities in the grading interfaces, which can theoretically cause stress concentrations that can adversely affect the effective material properties of FGLs. 

Such discontinuities caused by discrete step-wise grading have been recognized [29,30,31], and dealing with them in the design stage has been an active area of research. Researchers have attempted to use numerical modeling and machine learning in previous studies to estimate the loss of strength and to smooth the transition [32,33]. However, these studies are only concerned with two-dimensional truss-based lattices. The difficulty of three-dimensional force and topology analyses rises dramatically compared to 2D, and therefore, it is uncertain whether the proposed computational methods can be adapted effectively into 3D designs. Additionally, it is well-known that, unlike subtractive manufacturing, AM can cause the material properties of its products to differ significantly based on the printing process and relevant printing parameters. Stress concentration, the main reason researchers argue against aggressive grading, is almost always present in additively manufactured lattices due to rough surfaces [34,35,36,37]. Since computational models and theoretical calculations have not considered the manufacturing process-induced defects and printing parameters, the predicted discontinuity effects from these methods might not completely reflect reality. 

Given the challenge of incorporating printing defects and AM process parameters into computational models, experimental testing has been done to investigate the material properties of as-built FGL samples. Several previous studies have examined the compressive properties of truss-based FGLs using quasi-static experiments [38,39,40]. Furthermore, micro-computed tomography (micro-CT) has also been used extensively to examine the printing qualities of as-built samples, and researchers have confirmed that manufacturing defects are frequently present in SLM products, and these defects may have been the primary cause of strength reduction compared to computational models [41,42,43]. However, most studies focused on comparing FGLs and uniform lattices and concluded that FGLs have more desirable performances than uniform ones. Few studies have specifically designed FGL samples of the same solid volume fraction but use different gradients and compared the performances of FGL samples against each other. Therefore, the extent to which discrete unit cell grading-induced discontinuities can adversely affect the material properties of FGLs is still unknown. Further experimental testing is needed to determine whether the theoretical stress concentration caused by discrete unit cell grading is comparable to the already-observed stress concentration and material property loss caused by manufacturing defects and inaccuracies. 

This study investigates and compares the mechanical properties and manufacturing-induced defects for samples of the same volume fraction and hierarchical basic unit cells but different gradings and loading directions using compression testing, digital image correlation (DIC), and micro-CT imaging. Two discrete density-based gradings, linear and sharp grading, were used to design samples with different theoretical grading-induced discontinuities. The linearly graded sample designs are similar to several previous studies in that they all have step-wise linear gradients for truss-based lattices [18,39,44,45]. In contrast, the sharply graded sample designs incorporate larger volume fraction differences between graded layers arranged in a nonlinear gradient. We seek to combine the mechanical testing results and micro-CT imaging to examine the true effects of sharp grading when manufacturing defects are present. A total of 12 compression samples and 6 micro-CT samples were manufactured and tested from Ti64 powder using SLM with basic unit cells that have volume fractions ranging from 21% to 63%. The mechanical testing results were post-processed using DIC to obtain the mechanical properties of the FGL, which included true stress–strain curves, stiffness, yield stress, yield strain, and energy absorption capacities. Three types of struts, uniform struts shared in smooth grading and struts shared in sharp grading, were reconstructed and measured from micro-CT samples. By combining the results from mechanical testing and micro-CT measurements, we seek to experimentally quantify the potential effects of sharp grading on the mechanical properties of as-built FGLs. 

## 2. Methods

### 2.1. Mechanical Testing

#### 2.1.1. Hierarchical Design of FGLs

**Figure 1:**
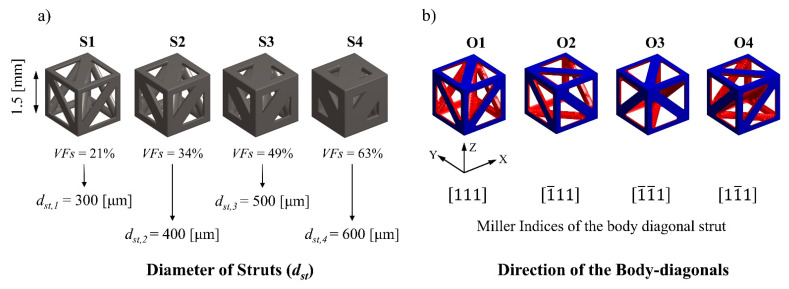
Proposed hierarchical basic unit cells of (**a**) four solid volume fractions, *VF_solid_*, and (**b**) four unit cell orientation types.

In this study, a hierarchical design process based on the assembly of a pre-defined set of basic unit cells was used for functional grading. Body Diagonal-Face Diagonal Cubic (BD-FDC) unit cells, according to the naming convention presented by Shi and Akbarzadeh [46], were selected as basic unit cells for assembling 2nd order hierarchical lattice structures. Each basic unit cell in the assembly took one out of four possible orientations and had a strut diameter assigned from four sizes ranging from 300 μm to 600 μm that corresponded to four different solid volume fractions from 37% to 79% for each sample (Figure 1). With different combinations of a total of 16 available types of basic unit cells (four unit cell orientations and four-volume fractions), functional grading of the lattice structure was achieved. A more detailed description of the hierarchical design process will be communicated in a separate publication.

**Figure 2:**
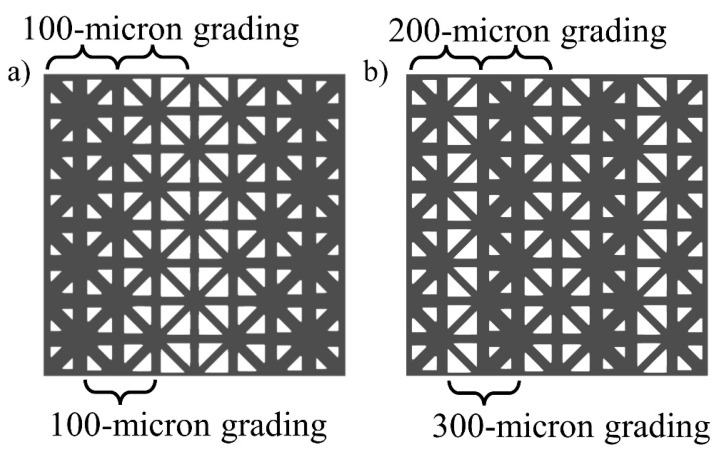
(**a**) Type L lattice with 100-micron thickness difference grading. (**b**) Type S lattice with 200-micron and 300-micron thickness difference grading. Both (**a**) and (**b**) are shown in the X-loading direction.

**Figure 3:**
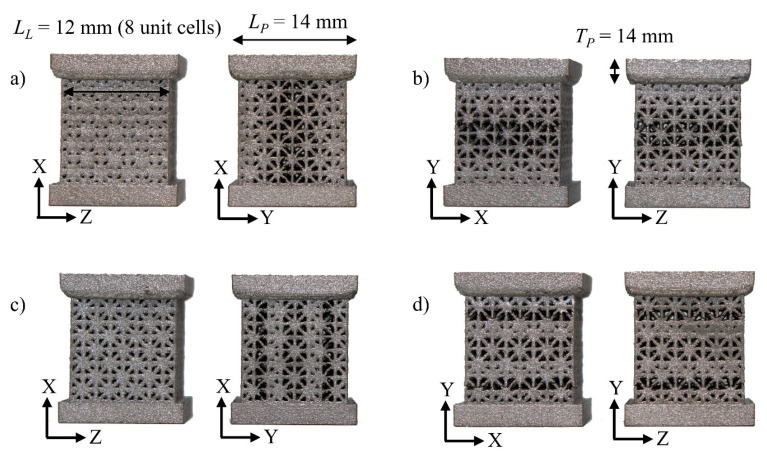
A total of 8 compression samples shown. Each sample group shows the front and the side views. For clarity, dimensions are distributed in different photos, but all the samples are manufactured to the same size. *L_L_*: Lattice body length. *L_P_*: Plate length. *T_P_*: Plate thickness. (**a**) X-direction type L, (**b**) Y-direction type L, (**c**) X-direction type S, and (**d**) Y-direction type S.

To compare the effects of grading on the mechanical properties of FGLs, we used the hierarchical design process for two types of grading: linear (or smooth) grading and sharp (or nonlinear) grading (Table 1). For linear grading, layers of unit cells with different volume fractions had maximum strut diameter differences of 100 μm, while for sharp grading, the strut diameter differences ranged between 200 and 300 μm (Figure 2). For clarity, linearly graded lattice bodies with smooth transitions are herein referred to as L lattices, whereas sharply graded lattice bodies are referred to as S lattices throughout the paper. The linear grading in this study also refers to stacking unit cells of discrete linear strut diameter difference [26,27,28], while sharp grading does not follow the linear step-wise strut diameter difference pattern. All lattice structures were graded in the same Y-direction, but they were tested in two directions to examine the anisotropy of the FGL. Two solid blocks were added to the top and bottom of the lattice structure in the direction of intended loading, which could be either X or Y-direction. One directional grading was chosen to eliminate off-axis stiffness terms from the elasticity tensor that could arise from interactions under different loading conditions, such as twisting or shearing during uniaxial compression. Due to the one-directional grading of the samples, the Z direction was not tested because it would duplicate the sample design of the X direction, as can be seen from Figure 3b,d, where the X-Y section view is exactly the same as the Z-Y section view.

While we recognize the potential of the Design of Experiment (DOE) in more complex scenarios involving additional variables like strut orientations and unit cell sizes, we effectively covered the design space using the One Factor at A Time (OFAT) method in our current study, with limited variables (grading pattern and loading direction, each with two values).

#### 2.1.2. Compression Sample Design and Manufacturing

Following the methodology of lattice grading described above, a total of 12 compression samples were designed and manufactured (Figure 3). This set included three samples in each of the four categories (x-direction type L, x-direction type S, y-direction type L, y-direction type S). The lattice bodies of the compression samples were cubic and contained 8 unit cells with a side length of 1.5 mm in each direction, thus having side lengths of 12 mm and a total of 512 unit cells. Top and bottom square plates with side lengths of 14 mm and thicknesses of 2.5 mm were added to the compression samples to ensure even contact when testing. The top plates were engraved with batch numbers for easy identification. Lattices loaded in x-direction were intended to resemble Voigt’s iso-strain conditions, while lattices loaded in y-direction were designed to resemble Reuss’s iso-stress condition. In each group of samples of the iso-strain or iso-stress condition, three samples had sharp grading, and the other three had smooth grading. The lattice structures were designed using an implicit geometry modeling framework developed in MATLAB R2020 (MathWorks, Natick, MA, USA) and nTop Version 3 (nTopology, New York City, NY, USA). The final designs were sent to 3D Systems’ On-Demand Manufacturing services (Rock Hill, SC, USA), now rebranded as QuickParts (Seattle, WA, USA, www.quickparts.com), for manufacturing with Ti64 alloy powder using Direct Metal Printing (DMP), an SLM process. Ti64 Grade 23 powder was used as a raw material with a layer thickness of 30 μm. The samples were heat-treated, and electrical discharge machining (EDM) was processed to a target surface roughness value of Ra = 2–4 μm.

#### 2.1.3. Experimental Testing and Data Processing 

Mechanical testing on 3D-printed samples designed for compression was used to experimentally emulate the mechanical properties of FGLs. The experiments were carried out on an MTS 810 machine with a maximum of 200 kips compression force, and data were gathered using the MTS 793 MultiPurpose TestWare software (https://www.mts.com/en/products/software-monitoring/793mpt). A metal block was placed above the samples to protect the MTS machine by preventing excessive stress that could cause the deformation of the contact surface due to the small surface area of the samples. A total of 12 compression tests were performed with one test per sample, and the loading rate was set to 0.5 mm/min in all tests. All samples were loaded monotonically until significant structural failures were observed or displacements exceeded 6 mm. The applied force was measured via a 200-kip load cell, and displacements were recorded using the internal displacement transducer of the hydraulic jack. 

The force-displacement data gathered from the MTS machine were processed using an in-house MATLAB code to estimate the material properties, including the initial linear elastic stiffness, yield stress, and yield strain. The maximum slope in the elastic region was found for each sample and extrapolated to the origin to represent elastic stiffness. The force-displacement response of each sample was normalized, which was then processed into the stress–strain curve (engineering and true). Two sets of parameters were used for normalization purposes. First, the vertical displacements (Δ*u_y_*) measured by the internal displacement transducer (DT) were divided by the total height of the sample (*H_T_*), which included the extra height due to the top and the bottom caps. The resulting engineering strain (*e*) was denoted by $\left(e_{1}=\Delta u_{y}/H_{T}\right)$. The measured reaction forces in the vertical direction (*F_y_*) were divided by the cross-sectional area of the lattice *A_L_*, resulting in engineering stress $\left(s=F_{y}/A_{L}\right)$. This normalization yielded an upper bound for the elastic modulus of the lattice structure, which was denoted by $\left(E_{1}=s/e_{1}\right)$. Secondly, the vertical displacements were normalized by the height of the lattice structure (*H_L_*), which excluded the height of the top and the bottom caps. This normalization resulted in a different engineering strain measure, which was denoted by $\left(e_{2}=\Delta u_{y}/H_{L}\right)$ and elastic modulus $\left(E_{2}=s/e_{2}\right)$. The data labeled with subscript 1 represents the material properties of the entire sample, including the top and bottom plates, and the data with subscript 2 represents the material properties of only the lattice part. Combined, these two sets of data represent the upper and lower estimations of the stiffness based on displacements recorded by MTS. Once engineering stress and strain (*s*, *e*) values were obtained, true stress and strain (*σ*, *ε*) were computed using $\varepsilon =ln\left(1+e\right)$ and $\sigma =s\left(1+\varepsilon \right)$. Elastic moduli based on true stress and strain were computed using $E_{t,1}=\sigma_{1}/\varepsilon_{1}$ and $E_{t,2}=\sigma_{2}/\varepsilon_{2}$. The 0.2% offset method was used to find the corresponding yield stresses (*σ_Y_*_,1_ and *σ_Y_*_,2_) and yield strains (*ε_Y_*_,1_ and *ε_Y_*_,2_) based on the true stress–strain curve of each sample. 

The testing processes were video recorded, and the videos were later used for Digital Image Correlation (DIC) processing and measuring lattice displacements externally. A cell phone camera (12 MP sensor, 77 mm equivalent f/2.8-aperture) was used for all samples, and an Olympus TG6 camera (12 MP sensor, 35 mm equivalent f/2.0-aperture, Olympus, Tokyo, Japan) was used in addition to cell phone recording on three samples to obtain high-resolution close-focus videos. Video recordings deemed suitable (9 of the 12) were used for DIC processing to measure the deformation of the lattice structure directly at the one frame-per-second rate. One video for a sharply graded Y-direction sample and two videos for linearly graded Y-direction samples were unsuccessful in DIC processing due to improper image focus. The pixel size for each footage was calibrated based on calibration patterns that were placed around the sample before starting the test. An opensource implementation of 2D Finite Element Global Digital Image Correlation (FE-DIC) in MATLAB [47] was used for DIC processing, and in-house MATLAB code was utilized for estimating the DIC-adjusted loading rates only for the lattice structures, excluding other deforming components. By incorporating the DIC-adjusted loading rates, we corrected the force-displacement results according to the vertical displacements of the lattice structure and calculated DIC-adjusted elastic modulus (*E_t_*_,DIC_), yield strength (*σ_Y_*_,DIC_) and yield strain (*ε_Y_*_,DIC_). Since DIC only tracks the deformation of the lattice parts, DIC-adjusted data only applies to the lattice part, excluding the top and bottom plates.

Additionally, the DIC-adjusted stress–strain curves were used to calculate the samples’ energy absorption capacities. For a compressed cellular structure, the energy absorbed per unit volume (*W_V_*) is the area under the stress–strain curve and can be calculated using Equation (1).

(1) $$W_{V}=\int_{0}^{\varepsilon_{a}}\sigma \left(\varepsilon \right)d\varepsilon$$

where $\varepsilon_{a}$ is the strain of the point at which the energy absorption is estimated, and $\sigma \left(\epsilon \right)$ is the stress at that point. The onset strains of densification (OSD) were calculated for all the X-direction samples by identifying the global maximum [48] when using the maximum efficiency method proposed by Li et al. [49]. The energy absorption efficiency parameter η(ε) is defined as the energy absorbed per unit volume (W_V_) at a given point divided by the stress at that point, given by Equation (2).

(2) $$\eta \left(\varepsilon \right)=\frac{1}{\sigma \left(\varepsilon \right)}\int_{0}^{\varepsilon_{a}}\sigma \left(\varepsilon \right)d\varepsilon$$

### 2.2. Micro-CT Imaging

#### Micro-CT Sample Design and Manufacturing

**Figure 4:**
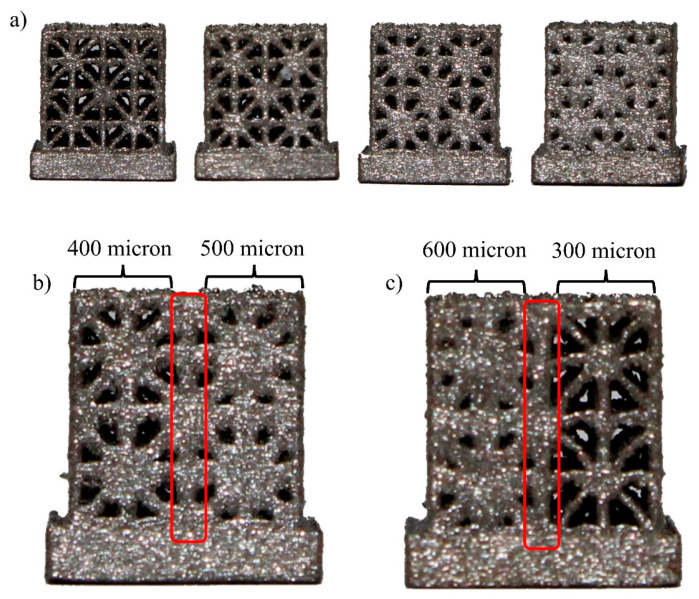
(**a**) Uniform strut thickness micro-CT samples from 300–600 microns with 100-micron interval. (**b**) One side view of the graded micro-CT sample, with the center strut (marked in red) shared by the 400-micron and 500-micron sides (linear grading). (**c**) Another side view of the graded micro-CT sample, with the center strut shared by the 600-micron and 300-micron sides (sharp grading).

A total of five micro-CT samples were designed and manufactured, including four samples of uniform strut diameters ranging from 300 microns to 600 microns with an interval of 100 microns (Figure 4a) and an additional sample that included transitions between unit cells of varying sizes, ranging from 100 microns for smoother transitions to 300 microns for sharper transitions (Figure 4b,c). Due to the limited X-ray power of the available desktop micro-CT scanner model and limited field of view, micro-CT specific samples were designed on a 4-by-4-by-4 grid using the same basic unit cells with side lengths of 1.5 mm, making them compact enough to fit inside the field of view of the scanner. A solid plate with a width of 8 mm and thicknesses of 2 mm was added at the bottom of each lattice design for marking and easy mounting onto the micro-CT platform. The micro-CT samples were manufactured using the same method as compression testing samples.

### 2.3. Micro-CT Procedure and 3D Reconstruction

Grayscale horizontal images obtained from micro-CT were used for visual inspection and 3D reconstruction. The samples were scanned using a Bruker SkyScan 1272 micro-CT scanner (maximum 100 μA and 100 kV, Bruker, Billerica, MA, USA). The samples were placed and fixed on a rotating platform using clay during scanning. Due to the different solid volume fractions, exposure times ranged from 2500 milliseconds to 3500 milliseconds. All scans used a 0.11 mm Cu filter to reduce noise. After scanning, the companion software for CT reconstruction was used to convert vertical X-ray images into horizontal slices. During this process, typical micro-CT post-processing correction methods such as alignment correction and ring artifact reduction were applied. The processed horizontal slices were used for visual inspection of manufacturing defects. They were also rebuilt into 3D bodies for strut measurements using the marching cube algorithm [50] in the form of STL files with the same pixel size as used for CT reconstruction. Geomagic Wrap 2017 (3D Systems) was then used to register the reconstructed geometries from micro-CT to the designed lattice structures, and the relevant affine transformation (i.e., rotation matrix) for perfect alignment of imaged models was extracted. With the rotation matrices, the original 3D bodies produced by the marching cube algorithm could be corrected to the right orientation and stored in the form of 3D volumes in MATLAB.

### 2.4. Micro-CT Measurements

**Figure 5:**
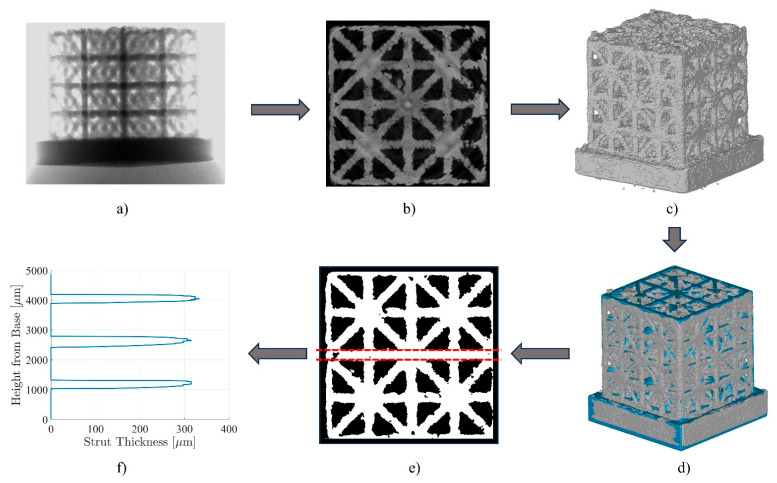
Process flowchart of micro-CT processing from scanning 3D-printed lattice samples to measuring the cross-sectional areas of horizontal struts. (**a**) X-ray image. (**b**) Processed CT image using the companion software. (**c**) 3D reconstructed model using marching cube algorithm via MATLAB. (**d**) Global registration of the 3D reconstructed model (grey) with the original design model (blue) via Geomagic 2017. (**e**) Strut thickness measurement of binarized slices via MATLAB. (**f**) Strut thickness measurements over all the slices in the vertical direction.

Cross-sectional areas of both uniform and graded struts, located at the transition between cells with different densities, were measured from the reconstructed 3D bodies. A MATLAB script was developed for counting the number of solid pixels in binarized 2D slices by sweeping along an axis of the stored 3D volume. Slices in the vertical direction were used for measurements due to the higher quality of images in the Z-direction, which was the original axis of rotation during scanning. The cross-sectional areas of struts were measured by finding the position of each strut and gradually expanding the upper and lower boundaries until the number of solid pixels dropped below a prescribed limit. At each expansion step, the algorithm computed the number of solid pixels that each boundary passed through within the width of the sample and compared it against void pixels. Each boundary stopped expanding until it passed through a target number of void pixels to account for the surface roughness and compute the average thickness of the measured strut, defined as half of the theoretical number of solid pixels along that row if the sample were smooth. In this way, the thickness of the horizontal struts could be measured for each valid 2D slice and integrated numerically for consecutive valid slices to compute the cross-sectional area of the strut (Figure 5). 

### 2.5. Statistical Analysis

One-way ANOVA tests, conducted using MATLAB, were utilized to compare various properties of type L and type S samples. These properties included stiffness values, yield stresses, and yield strains from the DIC-corrected values. Similarly, one-way ANOVA tests were performed on strut cross-sectional areas as measured from micro-CT images. However, for the energy absorption capacity curves of type L and type S samples, we relied on visual inspection, given the limitations of conventional statistical tests for variables of this type.

## 3. Results

### 3.1. Mechanical Testing Results

#### 3.1.1. Force-Displacement Responses

**Figure 6:**
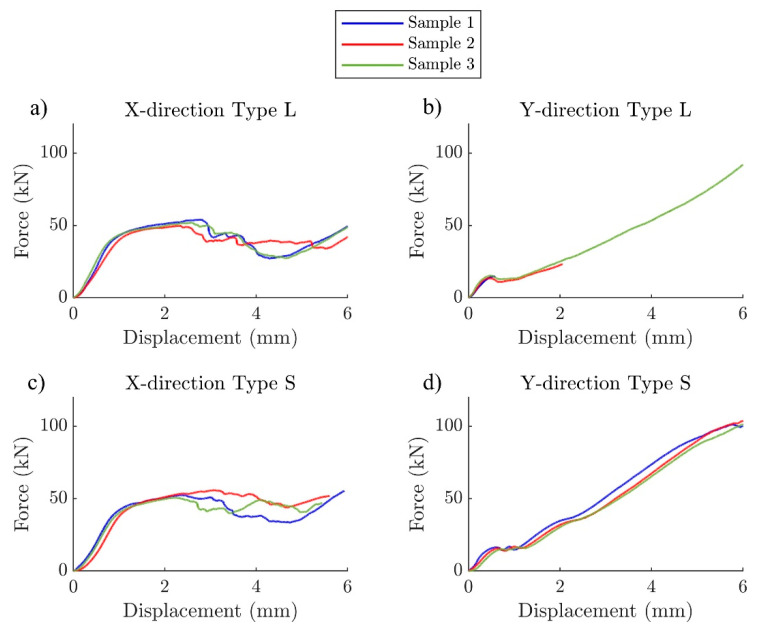
Unprocessed force-displacement data directly from MTS (unit conversion from inch to mm is done) for (**a**) X-direction type L, (**b**) Y-direction type L, (**c**) X-direction type S, and (**d**) Y-direction type S.

Force-displacement responses, as obtained from the MTS internal transducers, generally match well within each sample group (n = 3) over the entire loading range for all groups (Figure 6). It should be noted that technical difficulties with the video recording resulted in earlier stopping for tests on two samples of type L in Y-direction, thus displaying only a fraction of the entire compression testing compared to the other samples. However, since the tests were interrupted after the yield point, the stiffness and yield strength could still be calculated for these samples. It is clear from the force-displacement curves that the mechanical responses of these lattice designs are different depending on the loading directions. However, the mechanical responses of sharply and smoothly graded lattices appear similar in both loading directions. 

#### 3.1.2. Stress–Strain Curves

**Figure 7:**
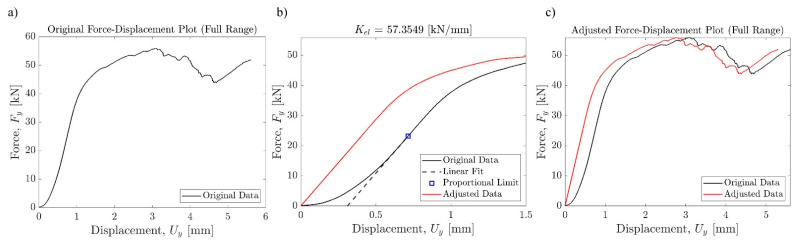
Post-processing of the raw force-displacement data from MTS. (**a**) Raw force-displacement curve. (**b**) Linearization of elastic region. (**c**) Comparison of raw and processed force-displacement curve.

**Figure 8:**
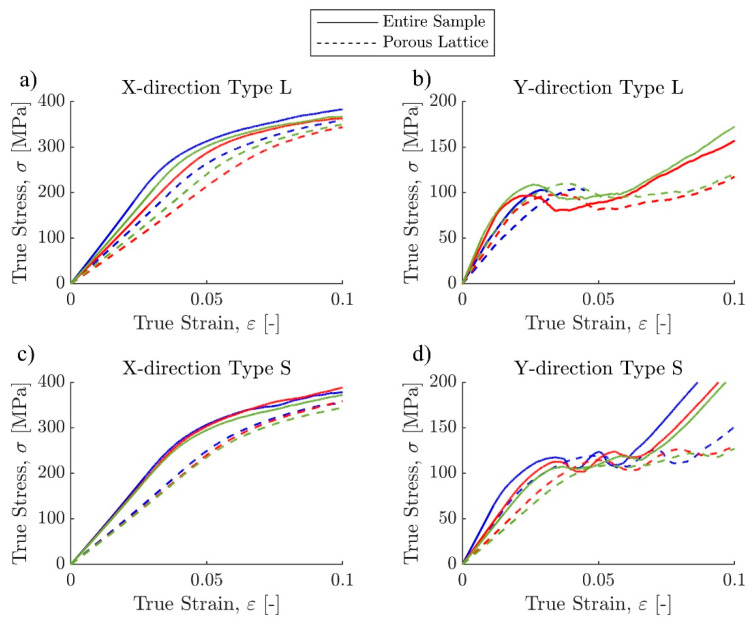
True stress–strain curves for (**a**) X-direction type L, (**b**) Y-direction type L, (**c**) X-direction type S, and (**d**) Y-direction type S samples processed from raw MTS data. Solid lines represent all the compression samples, including the top and bottom plates, and dashed lines represent only the lattice parts.

Stress–strain curves for all 12 samples were extracted from their force-displacement data using the post-processing technique explained in the methods section (Figure 7). The processed stress–strain curves show that the overall trend in stiffness within each sample group is consistent, but some differences can still be observed (Figure 8). Stress plateaus can be observed in both X-direction sample groups. The stiffness values of Y-direction samples were similar before yielding, but the linearly graded samples did not exhibit layer crushing behavior, while the sharply graded samples had two layer crushing behaviors immediately after initial yielding. Samples loaded in the Y-direction passed the yield point at lower displacements than the X-direction samples, with the stress–strain curves becoming nonlinear when the strain was around 1.5%, while X-direction samples did not yield until the strain reached at least 2.5% (Table 2). 

#### 3.1.3. DIC Tracking Results

**Figure 9:**
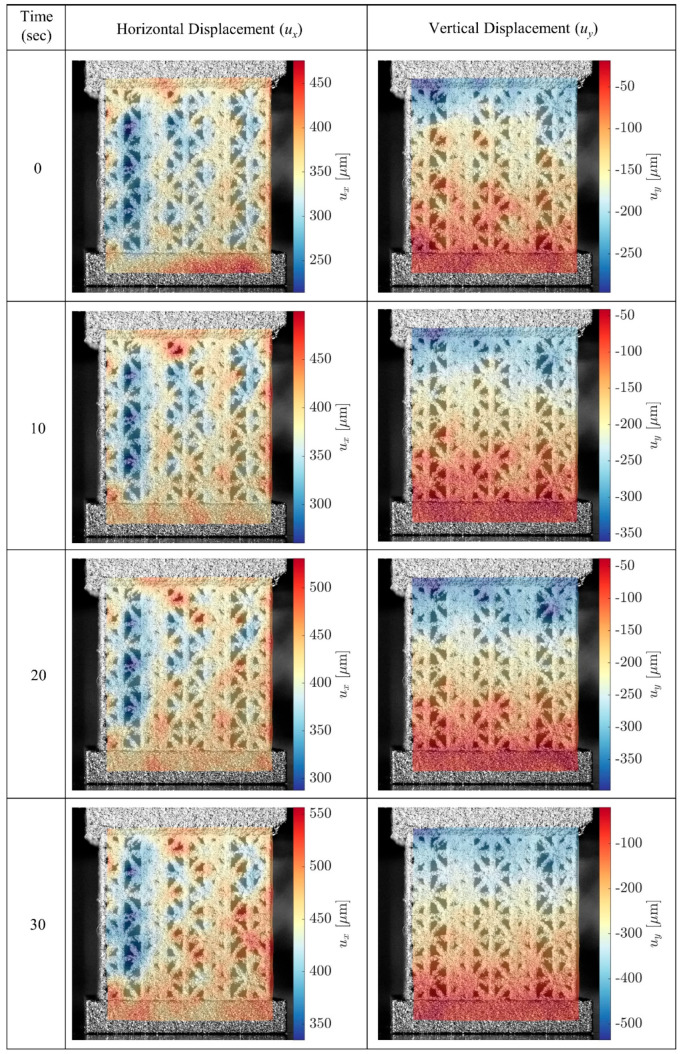
DIC processing snapshots for horizontal and vertical displacements in a 30-s interval (X-direction type S sample shown). Reproduced with permission from Solid Freeform Fabrication (SFF) Symposium, 2023, [51].

**Figure 10:**
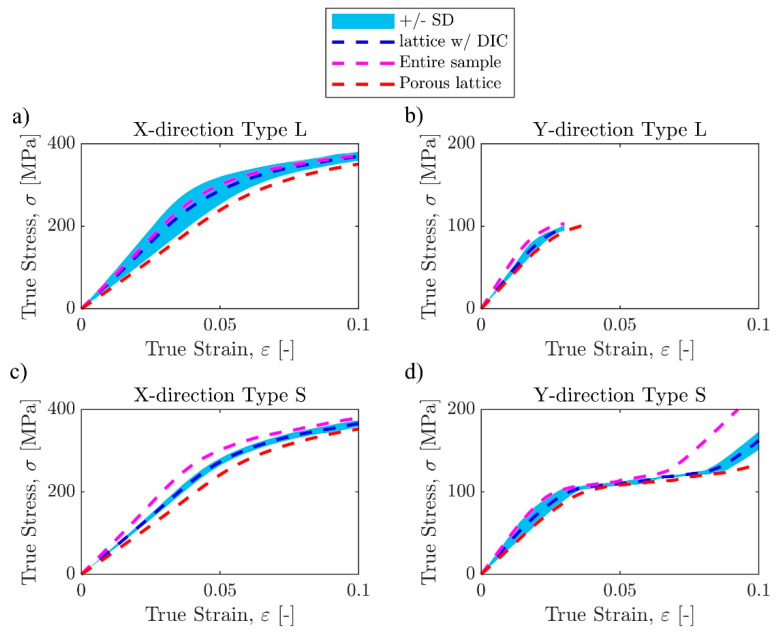
Average raw true stress–strain curves and DIC processed stress–strain curves with +/− one standard deviation. (**a**) X-direction type L. (**b**) Y-direction type L. (**c**) X-direction type S. (**d**) Y-direction type S.

**Figure 11:**
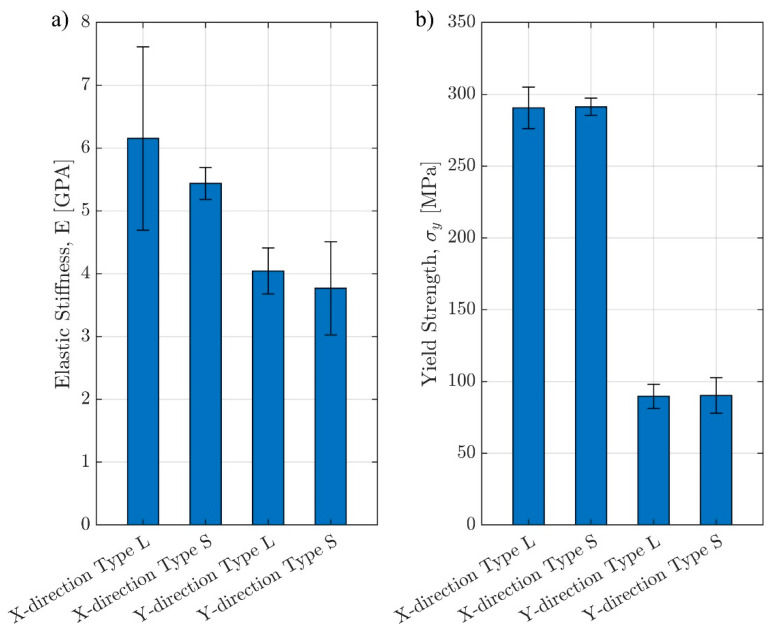
Comparison of (**a**) elastic stiffness and (**b**) yield strength by sample group. Error bars show standard deviations.

DIC successfully tracked the natural pixel patterns caused by surface roughness and outputting horizontal and vertical displacements (Figure 9) and surface strains (not shown here). After the updated vertical displacements were incorporated in calculating the stress–strain curves, it can be observed that for all samples, DIC-adjusted stiffness values are larger than the ones obtained by normalizing the internal DT measurements for the porous lattice structure, i.e., *E_t_*_,DIC_ > *E_t_*_,2_. Overall, the stress–strain responses computed from the DIC were comparable to those calculated from only the MTS measurements when averaging over three samples of each lattice design. The analysis of variations within each design showed that the stress–strain curves from the DIC generally would fall between those from MTS (Figure 10). The standard deviation was calculated using the data points from three samples within each sample group throughout the DIC-processable displacement range. For the sample groups with all three DIC processable videos, the displacement adjustment ratio from each video was applied to its corresponding sample individually. For the sample groups with less than three suitable videos, adjustment factors for the samples without valid videos were estimated as an average from the available correction ratios within the same sample group. The average stiffness values of type S samples are slightly smaller than type L samples in the same loading direction, but because of the rather substantial standard deviation, the differences are not large enough to produce statistical significance (*p* = 0.4466 for X-direction, *p* = 0.5976 for Y-direction). There are also no statistically significant differences in yield stress (*p* = 0.9365 for X-direction, *p* = 0.9510 for Y-direction) and yield strain (*p* = 0.5734 for X-direction, *p* = 0.6095 for Y-direction) between type S and type L samples in the same loading direction. Anisotropy is clearly observed, particularly in yield stress for the same grading type loaded in different directions, as the X-direction type L and type S samples have yield stresses around 290 MPa, while both Y-direction samples are around 90 MPa. Although anisotropy is less pronounced in stiffness than the yield stress, statistical significance still exists to support the observation, with the linearly graded samples having a weaker trend (*p* = 0.0712 for type L, *p* = 0.0210 for type S). These findings are summarized in Table 2 and Figure 11.

### 3.2. Energy Absorption Results

**Figure 12:**
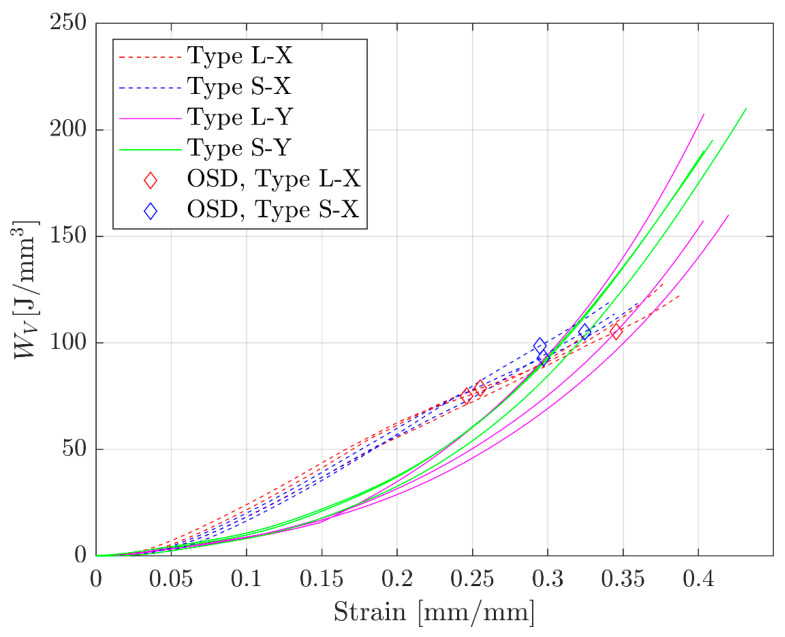
Energy absorption curves for all 12 samples and the densification strains for X-direction samples.

The energy absorption capabilities were similar between linearly and sharply graded samples in the same loading direction. Despite the lack of statistical tests, it is clear that the grading pattern does not affect the energy absorption capacities of the samples loaded in the same direction, as the curves from the two grading patterns overlap with each other. All the energy absorption curves are plotted along with the densification strains for X-direction samples (Figure 12). However, the maximum efficiency method does not apply to Y-direction samples, which will be discussed later. Despite two of the compression tests being interrupted for Y-direction type L samples, these two samples could be retrieved from the MTS machine and were tested again later. The energy absorption curves for these two samples are the combinations of the initial and the following tests. 

### 3.3. Micro-CT Results

#### 3.3.1. Additive Manufacturing Defects

**Figure 13:**
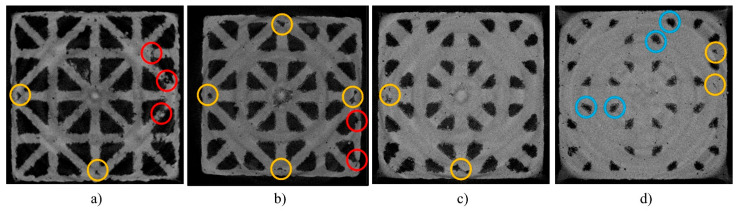
Reconstructed micro-CT slices for uniform samples with manufacturing-induced defects. Strut diameters are (**a**) 300 micron, (**b**) 400 micron, (**c**) 500 micron, and (**d**) 600 micron. Yellow circles: manufacture-induced defective pores. Red circles: Manufacturing-induced disconnected struts. Blue circles: loss of geometric conformity.

Additive manufacturing-induced defects were observed in horizontal grayscale images of all samples obtained from micro-CT (Figure 13). The most common defect is manufacture-induced defective pores (marked in yellow circles), observed in all strut thicknesses. At lower volume fractions, manufacturing-induced disconnected struts (marked in red circles) started to appear, potentially due to the strut thicknesses approaching the minimum recommended printing feature size (254 μm). At higher volume fractions, particularly the uniform 600-micron sample, designed void spaces (see as dark triangles in images) tended to lose geometric conformity, deforming from triangles into irregular shapes (marked in blue circles). 

#### 3.3.2. Dimensional Accuracy Measurements

**Figure 14:**
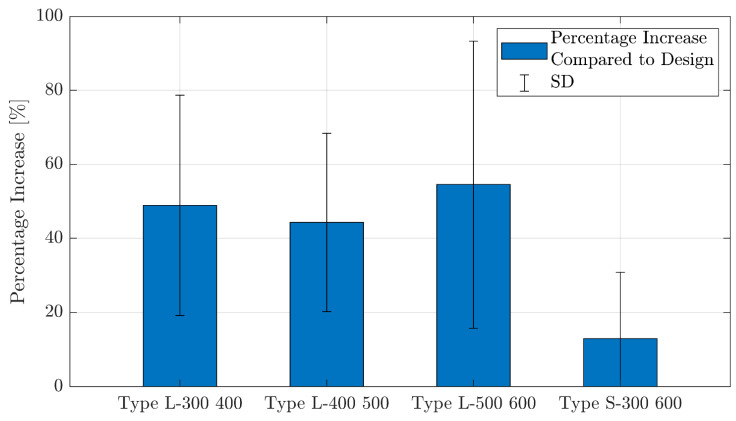
Comparison of percentage increase in strut cross-sectional areas between linear and sharp grading.

The dimensional accuracy measurements of horizontal struts revealed a 25 to 40% increase in cross-sectional areas (*A_st_*) of as-built samples compared to original designs for uniform and type L designs, which is significantly larger than an average of 13% increase for type S as-built samples (*p* = 0.0256 when comparing type L and type S designs), as shown in Table 3 and Figure 14. These differences in cross-sectional areas are equivalent to a 12 to 20% increase in strut widths for lattices with uniform density and type L grading and 6% larger strut widths for sharply graded struts. The reason why graded designs have fewer available struts is that the graded struts were all integrated into one sample. In contrast, the uniform designs have a total of 4 samples for different strut diameters, thus providing more data. 

## 4. Discussion

In this study, a total of 12 compression testing samples and 5 micro-CT samples were examined. The compression testing data from the MTS machine was processed in combination with DIC to produce stress–strain and energy absorption curves. The mechanical testing results show no statistically significant difference between the material properties of linearly graded and sharply graded hierarchical FGL samples, including stiffness, yield strength, yield strain, and energy absorbed per unit volume when the volume fractions of the samples are kept constant. Relatively large variations in mechanical properties within the samples of identical design groups were observed. These large intra-group variations are well-known in 3D-printed mesoscale lattice samples [52,53], which may be caused by manufacturing defects common for samples produced by SLM [54]. These large variations can be a cause for the lack of statistical significance. However, it is also worth noting that large variations appear in three out of four sample groups in this study, indicating that it is very hard to avoid them when using SLM printing. The micro-CT results show that the sharply graded strut has a significantly smaller percentage in cross-sectional area compared to design than linearly graded struts. Nonetheless, all three types of struts (uniform, linearly graded, and sharply graded) were printed larger than the design. The reconstructed micro-CT slices also confirmed multiple types of manufacturing defects, including defective pores, manufacturing-induced disconnected struts, and loss of geometric conformity. The results of this study suggest slight decreases in the elastic modulus of sharply graded Functionally Graded Lattices (FGLs) but do not indicate any adverse effects of sharp grading on the yield strength of FGLs. The minor reduction in the elastic stiffness of sharply graded FGLs may be attributed to observed disparities in the cross-sectional areas of linearly graded and sharply graded struts. Micro-CT measurements of the as-built cross-sectional areas of struts demonstrated that sharp transitions generally result in significantly smaller overall cross-sectional areas in the graded struts. Nevertheless, the marginal decreases in the elastic modulus due to sharp grading were deemed negligible when considering the substantial variations in elastic modulus within samples of the same type. Our findings reveal that the influence of 3D-printing defects on the elastic modulus outweighs the minor reductions attributable to sharp grading.

**Figure 15:**
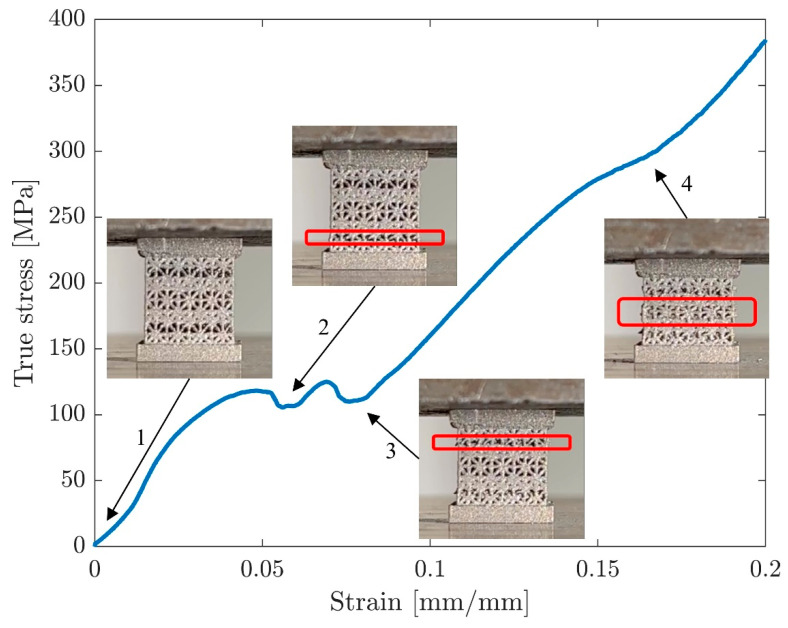
Three distinct layer collapse behaviors associated with local strain densification for a Y-direction type S sample. Red frames highlight individual local layer crushing.

The mechanical testing results are consistent with previous studies using the same material (Ti64) with similar volume fractions [15,55,56]. When comparing the mechanical behavior of graded lattices, Xiao and Song also found in their study that step-wise grading is not significantly different from continuous grading [19], which supports the conclusion of this study. Regarding the strain densification of Y-direction samples, previous studies have found similar results: When the lattice sample assembles Reuss’s iso-stress condition, it displays sequential layer collapse behavior [57]. In this study, this effect is more pronounced in sharply graded Y-direction samples (Figure 15). Since Y-direction type S samples have two separate layers of 300-micron unit cells and two 400-micron unit cell layers in the middle, three sequential layer collapses can be observed in each sample’s stress–strain curve. In each layer collapse, the sample experienced a local densification. The 500-micron and 600-micron unit cell layers can be treated as cell walls in these local densifications because of their significantly higher strength. The destruction of 500-micron and 600-micron unit cell layers happened towards the end of the experiment, just before or concurrently with the onset of true global densification. While this behavior is less obvious in Y-direction type L samples because the cell wall effect in local strain densification is minimized due to the smaller neighboring layer strut size difference in linear grading, the key material properties are still similar to sharply graded samples. The failure patterns for X-direction samples were diverse in cracking positions and orientations. This variability in failure patterns can be potentially attributed to inconsistencies in the internal truss structures and the surface roughness of the top and bottom plates, which may have caused uneven contact between the samples and the testing rig. The resulting uneven load distribution was much more impactful for X-direction samples only because their construction resembles the iso-stress condition. A comprehensive computational modeling analysis of these failure modes is ongoing and will be detailed in a subsequent manuscript.

Despite the difference in strut cross-sectional areas, linearly and sharply graded FGLs did not display statistically significant differences in mechanical properties. Micro-CT results show that the cross-sectional areas of all types of struts in the as-built samples were significantly larger than the original design. For uniform and linearly graded struts, the percentage increase in area decreases as the design strut diameter increases, indicating the possibility of a fixed increase in strut width. This can result from overhang structures commonly found in SLM-manufactured lattices [15]. Another possibility is that since the deviations in measured diameters are in the same order of magnitude as the printing layer thickness, the minimum feature size may be approaching the minimum printing resolution of the SLM process that was used (30 μm), causing the struts to have larger widths by about twice the printing resolution. Although sharply graded struts were found to be smaller in cross-sectional areas than linearly graded struts, two possibilities can lead to the similar mechanical properties exhibited by the samples of two grading types. Firstly, the roughness of the strut surface created by overhang structures and attached powders may have smoothed the discontinuity in sharp grading, thus reducing or eliminating the additional effects of stress concentration caused by more aggressive discontinuity. Secondly, the material properties are largely dependent on the weakest struts of the sample. Considering that micro-CT analysis has confirmed defective pores and manufacturing-induced disconnected struts, it is possible that these manufacturing-induced defects caused the samples of both grading patterns to fail well before the effects of predicted stress concentration in sharp grading become significant. Thus, it can be concluded that previous computational models may not be sufficient in accurately predicting the effects of sharp grading because these models do not incorporate the manufacturing process, but the printing defects and size deviation may be the dominating factors in determining the material properties of as-built samples.

This study had some limitations. The first limitation is the lack of control over printing parameters such as laser power, laser offset, scanning speed, cooling rate, etc. It is known that these parameters can significantly impact printing quality [58]. However, due to the lack of an in-house SLM printer, the sample designs could only be sent to external on-demand parts services. Nonetheless, the samples were printed following the standard practice in the field of orthopedic implants by using cloud manufacturing and were visually examined when received. The quality requirements were also communicated to the manufacturer. Samples with significant externally visible defects were reprinted. Next, only quasi-static testing was conducted in this study to determine the material properties of the lattice samples. Some material properties are better captured by other testing, such as bending and torsional. However, quasi-static compression testing is still a common approach in the study of various lattices [30,38,39]. Thirdly, this study’s DIC video and processing apparatus were not professional grade. The processed videos were repurposed recordings of the test rather than high FPS and resolution videos typically used for DIC processing, but since the goal of DIC processing in this study was to determine the relative displacement between the top and the bottom plates rather than examining surface strain, lower quality videos were acceptable. The sample size for each group of lattices is three, which is relatively small, but it is worth noting that the small sample size also reflects the real-world manufacturing situation of customized medical devices. The micro-CT machine that was used in this study had limited power; thus, the boundary between attached powder and actual struts was not visually distinctive, but the latest micro-CT slices scanned with a more powerful machine thanks to external support revealed that by identifying the correct threshold, the binarizing process was able to screen and eliminate most of the attached powder before measuring. The fourth limitation of the study is that the actual surface roughness of the lattice samples could not be measured using a white light interferometer. This was due to the inherent void spaces in the truss-based lattice structures of our samples. Consequently, only the target value of Ra = 2–4 μm was reported. Nonetheless, the findings of this study were independent of the surface roughness of the samples. Surface roughness may be important for osseointegration in implant applications, but this property is out of the scope of this study. Finally, this study only measured horizontal struts, which are mostly affected by the overhang effect. However, since the strut cross-sectional area comparison was performed consistently using horizontal struts from the lattice samples, the conclusion does not change. 

## 5. Conclusions

In this study, we focused on evaluating the impact of grading pattern sharpness on the mechanical properties of lattice structures manufactured via SLM with Ti64 powder under consistent volume fractions and loading conditions. We discovered that the sharpness of the grading pattern does not significantly affect key mechanical properties such as elastic stiffness, yield strength, and energy absorption in graded lattice structures. This finding holds true despite the variations in strut diameters, as observed in micro-CT analyses. Further, our dimensional accuracy analysis indicated reduced strut cross-sectional areas in samples with sharp grading patterns. Micro-CT imaging also revealed the presence of manufacturing-induced defects, including defective pores and disconnected struts. These observations suggest that the primary factors affecting the mechanical properties of the tested lattice structures are these manufacturing defects rather than the stress concentrations typically associated with aggressive grading patterns.

## Figures and Tables

**Table 1:** Linear and sharp discrete grading within functionally graded lattice structures designed based on the hierarchical concept.

Linear (L) Grading	Sharp (S) Grading	
100-Micron	200-Micron	300-Micron
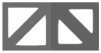	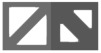	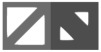
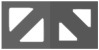	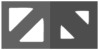	
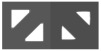		

**Table 2:** Effective material properties, i.e., elastic stiffness (*E_t_*), yield stress (*σ_Y_*), and yield strain (*ε_Y_*) of test samples as obtained from MTS data (∙)_1_ and (∙)_2_ and DIC-adjusted data (∙)_DIC_. Reproduced with permission from Solid Freeform Fabrication (SFF) Symposium, 2023, [51].

Grading Type (Loading Direction)	Sample Number	*E_t_*_,1_ [GPa]	*E_t_*_,2_ [GPa]	*E_t_*_,DIC_ [GPa]	*σ_Y_*_,1_ [MPa]	*σ_Y_*_,2_ [MPa]	*σ_Y_*_,DIC_ [MPa]	*ε_Y_*_,1_ [-]	*ε_Y_*_,2_ [-]	*ε_Y_*_,DIC_ [-]
Type L (X-dir)	1	7.66	5.30	6.84	275.5	282.9	281.4	0.039	0.056	0.043
	2	5.82	4.03	4.48	297.4	312.5	307.1	0.053	0.079	0.066
	3	6.56	4.53	7.14	290.8	301.1	282.8	0.046	0.068	0.041
Average (SD)		6.68 (0.92)	4.618 (0.64)	6.15 (1.46)	287.9 (14.9)	298.9 (14.9)	290.4 (14.4)	0.046 (0.0071)	0.068 (0.011)	0.050 (0.014)
Type L (Y-dir)	1	4.99	3.45	3.85	81.9	77.7	81.0	0.018	0.025	0.023
	2	6.05	4.18	4.46	88.4	87.5	89.8	0.016	0.022	0.022
	3	4.56	3.19	3.80	101.1	100.1	97.9	0.024	0.033	0.027
Average (SD)		5.21 (0.76)	3.61 (0.50)	4.04 (0.36)	90.4 (9.7)	88.4 (11.1)	89.6 (8.4)	0.019 (0.004)	0.027 (0.0056)	0.024 (0.0031)
Type S (X-dir)	1	6.80	4.76	5.72	288.6	297.9	290.4	0.044	0.064	0.052
	2	6.57	4.54	5.35	291.1	301.8	297.6	0.046	0.068	0.057
	3	6.48	4.48	5.23	280.0	289.5	285.6	0.045	0.066	0.056
Average (SD)		6.62 (0.16)	4.60 (0.14)	5.43 (0.25)	286.5 (5.8)	296.4 (6.3)	291.2 (6.0)	0.045 (0.0009)	0.066 (0.0019)	0.055 (0.0025)
Type S (Y-dir)	1	5.57	3.89	4.59	92.9	91.4	88.7	0.018	0.025	0.021
	2	4.04	2.79	3.16	102.1	102.2	103.2	0.027	0.038	0.034
	3	3.58	2.47	3.54	100.3	101.2	78.5	0.030	0.042	0.024
Average (SD)		4.40 (1.04)	3.06 (0.74)	3.76 (0.74)	98.5 (4.8)	98.3 (5.9)	90.1 (12.4)	0.025 (0.0059)	0.035 (0.009)	0.026 (0.007)

**Table 3:** Measured Strut Cross-sectional Areas. U: uniform. L: linear grading. S: sharp grading.

	U-300	U-400	U-500	U-600	L-300~400	L-400~500	L-500~600	S-300~600
Design *A_st_* (mm^2^)	0.071	0.126	0.196	0.283	0.098	0.161	0.240	0.177
Average (SD) Measured *A_st_* (mm^2^)	0.099 (0.084)	0.177 (0.0068)	0.261 (0.0177)	0.358 (0.1267)	0.146 (0.0028)	0.232 (0.0063)	0.370 (0.0011)	0.200 (0.0308)
Sample Size (n struts)	9	9	9	8	3	3	2	2
Oversize Percentage	40.49%	40.50%	32.70%	26.64%	48.92%	44.28%	54.52%	12.95%

## Data Availability

Data is contained within the article.
